# Fire Phenomena of Rigid Polyurethane Foams

**DOI:** 10.3390/polym10101166

**Published:** 2018-10-19

**Authors:** Martin Günther, Alessandra Lorenzetti, Bernhard Schartel

**Affiliations:** 1 Bundesanstalt für Materialforschung und-prüfung (BAM), Unter den Eichen 87, 12205 Berlin, Germany; martin.guenther2@bam.de; 2 Department of Industrial Engineering, Padova University, v. F. Marzolo 9, 35131 Padua, Italy; alessandra.lorenzetti@unipd.it

**Keywords:** polyurethane, rigid foams, fire behaviour

## Abstract

Rigid polyurethane foams (RPUFs) typically exhibit low thermal inertia, resulting in short ignition times and rapid flame spread. In this study, the fire phenomena of RPUFs were investigated using a multi-methodological approach to gain detailed insight into the fire behaviour of pentane- and water-blown polyurethane (PUR) as well as pentane-blown polyisocyanurate polyurethane (PIR) foams with densities ranging from 30 to 100 kg/m^3^. Thermophysical properties were studied using thermogravimetry (TG); flammability and fire behaviour were investigated by means of the limiting oxygen index (LOI) and a cone calorimeter. Temperature development in burning cone calorimeter specimens was monitored with thermocouples inside the foam samples and visual investigation of quenched specimens’ cross sections gave insight into the morphological changes during burning. A comprehensive investigation is presented, illuminating the processes taking place during foam combustion. Cone calorimeter tests revealed that in-depth absorption of radiation is a significant factor in estimating the time to ignition. Cross sections examined with an electron scanning microscope (SEM) revealed a pyrolysis front with an intact foam structure underneath, and temperature measurement inside burning specimens indicated that, as foam density increased, their burning behaviour shifted towards that of solid materials. The superior fire performance of PIR foams was found to be based on the cellular structure, which is retained in the residue to some extent.

## 1. Introduction

Rigid polyurethane foams (RPUF) are widely used as an insulation material in the construction industry, for refrigeration, and in the pipe and tubing industry. Due to their extremely low thermal conductivity, RPUFs typically offer excellent insulating properties and outperform other commercially available insulating materials [1,2]. The reason for these unique characteristics is their cellular structure. Unfortunately, this structure results in poor fire behaviour. The time to ignition for thermally thick combustible materials is proportional to their thermal inertia, which is described as the product of thermal conductivity (λ), density (ρ) and heat capacity (c) [3,4]. Since λ and ρ are typically very low for foams, their thermal inertia is low. Hence, for a given heat flux the surface temperature of a cellular polymer rises more rapidly than for high values of λρc [5]. Because of their low thermal inertia, which benefits not only their ignitability but also their flame spread, RPUFs are considered hazardous [6,7,8].

Thus, a great deal of effort has been spent in investigating flame retardants for RPUFs [9,10,11,12,13,14,15,16,17], and recently several novel phosphorous FRs were tested [18,19,20,21,22,23]. A series of more detailed investigations focused on the fire phenomena of flexible polyurethane foams was performed, since they exhibit even more complex fire behaviour including structural collapse and the formation of pool fires [24,25,26].

However, a detailed understanding of the fire phenomena of RPUF is still lacking. Investigations focused on the fire phenomena are extremely rare. A profound comprehension of the processes taking place during combustion is the foundation for developing flame-retardant approaches [27]. Therefore, a set of foams with systematically varied properties was prepared. RPUF was obtained using water and pentane as blowing agents. Besides RPUF, a rigid polyisocyanurate foam was examined, since these foams are known for their higher stability from a thermodynamic point of view [28]. Using a multi-methodological approach, the complex interaction of fire phenomena was studied in detail. Cone calorimeter measurements provide insight into fire behaviour under forced-flaming conditions. Sample-holders were equipped with glass windows to monitor the materials’ response to cone calorimeter test conditions. Additionally, thermocouples were inserted into the specimens, giving information about the temperature gradient inside of the sample and the temperature of the pyrolysis zone during combustion. Changes in the morphology and thickness of the pyrolysis zone were examined using scanning electron microscope images of cross sections of quenched foam specimens. The determination of the oxygen index and the use of thermogravimetry measurements completed the investigation. A more detailed insight into the complex burning behaviour of RPUFs is delivered and potential relations between material properties and resulting fire behaviour are shown.

## 2. Materials and Methods

### 2.1. Materials

To gain a comprehensive overview of the burning behaviour of RPUF, a set of foams with well-defined properties was prepared as listed in Table 1. The samples were prepared at the Department of Industrial Engineering at Padova University in Padua, Italy. All examined materials were divided into three groups, namely pentane-blown polyurethane foam (PUR-P), water-blown PUR (PUR-H), and pentane-blown polyisocyanurate-polyurethane foam (PIR-P) and named indicating the density of the material. Different blowing agents were chosen in order to determine their influence on the fire behaviour of the foams. Every group of materials was tested in different densities, since density is the most important factor controlling the properties, is known to have a distinct influence on other characteristics of the foam [29], and can be varied without fundamentally changing the chemical composition of the material.

The raw materials employed in formulating the foams were two polyester polyols (Isoexter 4530 and 4537, Coim Italia, Offanengo, Italy) with nOH of 510 and 350 mg KOH/g, respectively, and one polyether polyol (Isoter 842G) with nOH of 160 mg KOH/g, all supplied by Coim Italia (Offanengo, Italy). Their weight ratio was kept constant (2:2:1) for all formulations. Polymeric MDI (methane diphenyl diisocyanate): Voranate M600 (Dow Chemicals) with NCO% = 30.5, average functionality = 2.8 and viscosity at 25 °C = 600 mPa·s was used. Dimethyl cyclohexylamine (DMCHA, Polycat 8, Evonik, Essen, Germany) and pentamethyl diethylenetriamine (PMDETA, Polycat 5, Evonik, Essen, Germany) were used for polyurethane (PUR) polymerization; in the case of polyisocyanurate (PIR) preparation the catalyst Dabco K15 (potassium-octoate in diethylene glycol) was also employed. The surface-active agent was Tegostab B 8469. Both the catalysts and the surface-active agent were generously supplied by Evonik (Essen, Germany). The foams were blown with a mixture of iso-and cyclo-pentane (70/30) or water. The amount of blowing agent was varied to obtain different densities ranging from 30 to 100 kg/m^3^. Using these raw materials, both PUR and PIR foams (with a constant NCO index of 110 and 300, respectively) were prepared.

### 2.2. Sample Preparation

The preparation of polyurethane and polyisocyanurate foams was carried out in two steps. Firstly, the formulated polyols (i.e., polyols mixture, catalysts, surfactant and blowing agent) were prepared and mixed; afterward, isocyanate was added by mechanical stirring for 15 s, and then the mixture was poured into a mould to produce foams. After preparation, the foams were kept in an oven at 70 °C for 24 h to complete the polymerization reaction and then cut to obtain specimens for characterization.

### 2.3. Characterization of Density, Compression Strength, and Thermal Conductivity

Apparent foam densities were measured according to ISO 845; compression strength was measured according to ISO 844 in parallel rise direction. Thermal conductivity was evaluated according to ISO 8301 by means of a heat flow meter apparatus (Holometrix Micromet Lambda 2300 V, Netzsch, Selb, Germany), at a mean temperature of 10 °C with a temperature difference between plates of 20 °C. All foams were tested one day after preparation to prevent any effect related to the ageing of the materials.

### 2.4. Specific Heat Capacity

The specific heat capacity at 25 °C was determined on a DSC 204 F1 (Netzsch, Selb, Germany) according to ISO 11357-4. A heating rate of 10 K/min was used and measurements were carried out under nitrogen atmosphere using a flow rate of 50 mL/min. Specimens were prepared by pressing the samples in a hydraulic press with a force of 8 t for 1 min and punching out. All measurements were repeated five times and averaged for each material. The standard deviation for each value was 5% or less.

### 2.5. In-Depth Absorption

The heat absorption of the foams was tested in a special experimental set-up proposed by [30]. Foam samples approximately 10 × 10 × 1 mm^3^ in size were pressed in a hydraulic press with a force of 8 t for 1 min and then measured with the setup described in [31,32]. Then, the absorption coefficient was calculated as shown in [30]. The dependency between the intensity and the absorption coefficient can be written as:

(1) $$\ln\left(\frac{I}{I_{0}}\right)=2\ln\left[\left(1-R\right)\right]-aS,$$

where I is the intensity after passing through material with a thickness of S, $I_{0}$ is the intensity prior to passing through the material, *R* is the average total reflectivity, and a is the absorption coefficient. When plotting $-\ln\left(\frac{I}{I_{0}}\right)$ over S, the curve has a slope of a [30]. The apparent absorption coefficient a was derived from the slope of each measured thickness from the origin to each point measured. 

### 2.6. Fire Behaviour

RPUF specimens 100 mm × 100 mm × 50 mm in size were tested according to ISO 5660 with a cone calorimeter from Fire Testing Technology (East Grinstead, UK). Different approaches for a suitable sample thickness of foams were reported, since the main disadvantage of such a thick sample is the decreasing heat flux with increasing distance between the cone heater and sample surface during combustion [24,33]. The cone calorimeter has some limitations regarding the measurement of sample with a low density and therefore low weight, low fire load, and short burning time [23,34] which lead to increasing uncertainty [35]. Thus, a thickness of 50 mm was chosen to obtain a sufficient sample mass. Specimens were conditioned in a climatic chamber for 48 h at 23 °C and 50% relative humidity prior to testing. The measurements were carried out with a heat flux of 50 kW/m^2^ and a separation of 25 mm. To protect the specimens’ sides and reduce unrepresentative edge burning, all samples were wrapped in aluminium foil.

Time to ignition ($t_{ig}$), heat release rate (HRR), peak heat release rate (PHRR), time to PHRR ($t_{PHRR}$), total heat released (THR) total mass loss (TML), and effective heat of combustion (EHC) calculated by THR/TML were evaluated. The results reported are the averaged values. The values for THR and TML were taken at flameout, which is defined as the extinguishment of visible yellow flames. Each measurement was repeated at least two times. The results were reproducible within 10% for all investigated results.

### 2.7. Temperature Measurement within Burning Foam

To measure the temperature development inside the foams during combustion, cone calorimeter samples were equipped with thermocouples in a vertical orientation at depths of 10, 20, and 30 mm from the surface directed toward the cone heater. Depths greater than 30 mm were not considered. Due to the foam consumption by the fire, the distance between cone heater and sample surface increases during the test leading to a decrease in heat flux. The decline of heat flux was found to be 11% for a sample of a thickness of 39 mm [5,24].

Thermocouples were inserted from the bottom and placed on the perimeter of a circle with a radius of 20 mm from the centre of the specimen, since this area is reported to have a uniform heat flux even at greater distances from the cone heater [36]. Each foam sample was equipped with six 0.5 mm type-K sheathed omega thermocouples, of which the ones opposite from each other were always inserted to the same depth. Therefore, the temperature for each depth was recorded twice per measurement. For each temperature graph, the heating rates and their maxima were determined. The averaged results are reported.

### 2.8. Quenching of Burning Foam

To allow investigation of the morphology in the area of the pyrolysis zone, specimens were quenched at the time point of 50 wt % mass loss in the cone calorimeter test. Therefore, burning samples were taken out of the cone calorimeter and dipped in liquid nitrogen to stop decomposition and freeze the state of the specimen at this particular time point. Following this, cross sections were taken and investigated further.

### 2.9. Limiting Oxygen Index

Flammability (reaction to small flames) was examined by means of the limiting oxygen index (LOI) according to ISO 4589. A specimen size of 100 mm × 10 mm × 10 mm was used. All specimens were conditioned in a climatic chamber for 48 h at 23 °C and 50% relative humidity prior to testing.

### 2.10. Thermogravimetric Analysis

For thermogravimetric analysis (TG), a TG 209 F1 Iris (Netzsch, Selb, Germany) was used. All materials were pyrolyzed under nitrogen with a flow of 30 mL/min at a heating rate of 10 K/min. TG was performed under pyrolytic conditions to simulate the lack of oxygen in a laminar flame. A sample size of 5 mg was used, and the samples were provided as powder in a ceramic crucible. All foams were milled under liquid nitrogen in a CryoMill (Retsch, Haan, Germany). For every material TG was done at least twice, the results reported are the averaged values. TG as well as differential TG (DTG) results are shown in this paper. The temperature of 5 wt % mass loss marks the beginning of decomposition and is hereafter referred to as T_95%_.

### 2.11. Characterization of Morphology

Cross sections of the undamaged foams and the quenched cone calorimeter samples, and the surface morphology of the cone calorimeter residues, were investigated with a scanning electron microscope (SEM). Images were taken using a EVO A10 (Zeiss, Jena, Germany). An acceleration voltage of 15 kV was used, and gold sputtering was applied to reduce image degradation due to charging effects. Cell wall thickness and strut size were measured by image analysis. For visual investigation of the pyrolysis zone, macro photographs were taken with a digital single-lens reflex camera using a macro lens with a focal length of 60 mm.

## 3. Results and Discussion

### 3.1. Foam Characterization

**Figure 1:**
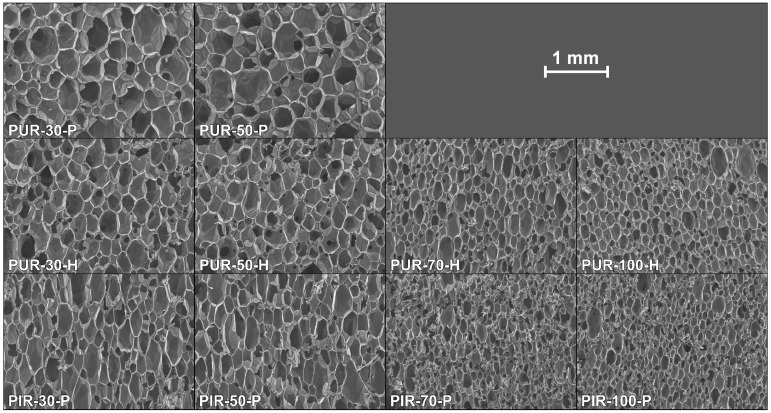
SEM micrographs of the cell structure of all tested materials.

SEM micrographs of each foam’s cross sections were taken at a magnification of 100 and are displayed in Figure 1. All materials show a homogeneous cell structure as well as mainly closed cells. This is typical for rigid polyurethane foams and desirable for most purposes since closed cell walls cause not only low water absorption and low moisture permeability but also the retention of the blowing agent, which is responsible for their low thermal conductivity. RPUF with a density of approximately 30 kg/m^2^ often has a closed cell content of 85–95% [1]. The investigated foams show rather homogenous cell sizes, which increase with decreasing foam density. Furthermore, different foams have comparable cell morphology at similar densities, rendering them comparable when referring to their burning behaviour.

Table 2 lists the results for thermal conductivity and compression strength. The thermal conductivity of closed cell foams depends on the density, the cell size, and the gas inside the cells of the foam and is not a linear relation. In fact, the thermal conductivity is determined by the thermal conductivity of the solid and of the gas as well as the radiation heat transfer from between the cell walls [37]. The data reported in Table 2 show that thermal conductivity generally tends to have a minimum in the range of 50–70 kg/m^3^. This is in agreement with the literature [37] and due to an increase of solid conduction, a decrease of radiation contribution, and a slight decrease of gas conduction with increasing density. In the literature it was reported that pentane-blown RPUF exhibits lower thermal conductivity than water-blown RPUF [38]. This was confirmed by the measurements. Common RPUFs used for construction have a density around 30 kg/m^3^, and their thermal conductivity can be as low as 24 mW/m·K [2]. Since the formulation of the foams investigated in this study was not optimized in regard to their thermal conductivity, λ is increased compared to commercial systems. Due to the increased stability of higher-density foam structures, the compressive strength increased with density [1]. While PUR-P and PUR-H exhibited similar values for compressive strength at the same densities, PIR-30-P had a significantly increased compressive strength of 348 kPa, compared with 195 kPa for PUR-30-P and 170 kPa for PUR-30-H. However, the compressive strength at a density of 100 kg/m^3^ was similar for PUR and PIR foams.

### 3.2. Pyrolysis Analysis and Limiting Oxygen Index

**Figure 2:**
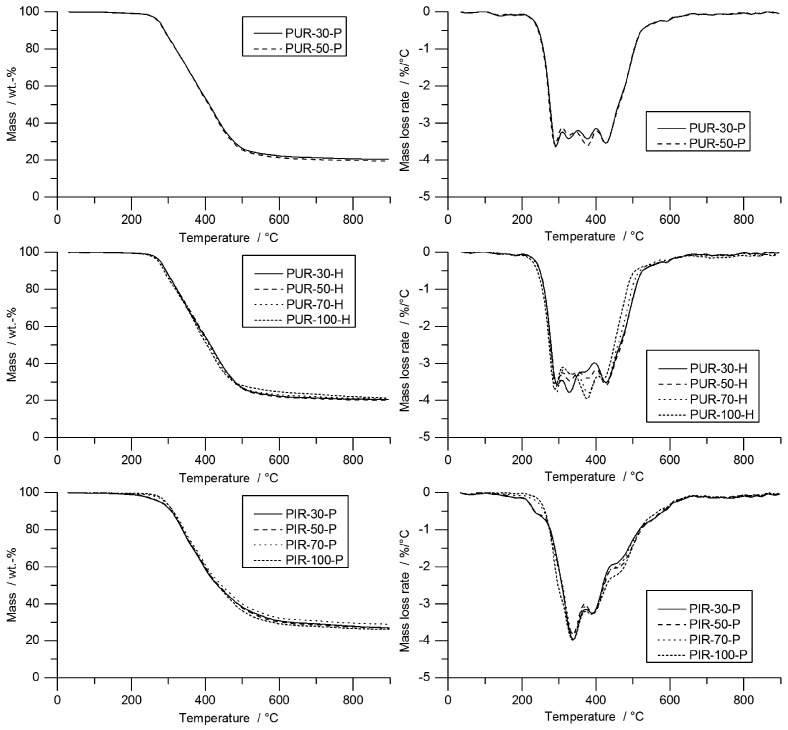
TG results of PUR-P, PUR-H, and PIR-P foams.

In TG, all materials showed their main decomposition step between 250 and 500 °C. The TG and mass loss rate (MLR) curves of PUR-P and PUR-H foams are shown in Figure 2. Since all foams were measured as powder, the density of the initial materials had no considerable influence on pyrolysis behaviour.

TG results are shown in Table 3. PUR-P and PUR-H exhibited similar decomposition behaviour, with a broad peak between 250 and 500 °C and four smaller local maxima. For comparison, only the first local peak was considered. Temperatures for the first peak ranged from 290 to 296 °C. All foams had a residue of approximately 20 wt %, and the T_95%_ ranged from 274 to 281 °C; the pyrolysis temperature (T_max_) was between 290 and 296 °C.

PIR-P foams showed decomposition behaviour with one major decomposition step around 340 °C. The increased thermal stability of PIR foams is caused by the isocyanurate ring structure [39]. The decomposition of PIR-P started in the range of 279 to 293 °C and increased slightly with density. PIR-P generally yielded a higher amount of residue: 27.4 wt % on average.

Table 3 contains the results of limiting oxygen index (LOI) measurements for all foams that were tested. Within every group of materials, the LOI increased with increasing density, although the effect is small. Indeed, the influence of density is limited to 1.2 up to 1.6 vol % and was related to the increased thermal inertia and the better char yield of higher-density foams. The LOI of PUR-30-H increased slightly, 20.1 vol % compared to 19.5 vol % for PUR-30-P, which is probably the consequence of using a non-flammable blowing agent. Due to the higher thermal stability of PIR-P foams, LOI increased by approximately 2 vol %, ranging from 21.5% to 23.1%. 

### 3.3. Fire Behaviour

#### 3.3.1. Time to Ignition

Due to the cellular structure of foams and the low thermal inertia this entails [40,41], the $t_{ig}$ of all materials measured with the cone calorimeter was 4 s or less (Table 4). A slight increase in $t_{ig}$ was measured for increasing densities, which was not significant, but was related to the increased thermal inertia of foams with higher density. In general, $t_{ig}$ for thermally thick materials can be described as [4,42]:

(2) $$t_{ig}=\frac{\pi }{4}\lambda \rho c\frac{{\left(T_{ig}-T_{0}\right)}^{2}}{{\dot{q}}_{ext}^{\prime \prime 2}-CHF},$$

where $T_{ig}$ is the ignition temperature of the foam, $T_{0}$ the ambient temperature, ${\dot{q}}_{ext}^{\prime \prime }$ the external heat flux, and CHF the critical heat flux that is necessary for ignition of RPUF in the cone calorimeter. Measured heat capacity as well as calculated and measured $t_{ig}$ for all materials is shown in Table 4. The following parameters were used to calculate $t_{ig}$: $T_{ig}$ = 305 °C [40]; $T_{0}$ = 20 °C; ${\dot{q}}_{ext}^{\prime \prime }$ = 50 kW/m^2^; CHF = 23 kW/m^2^ [4].

The calculated $t_{ig}$ underestimate those measured by one order of magnitude. The reason for this is that the formula for estimating $t_{ig}$ has some approximations, e.g., it assumes that the entire amount of incident radiation is absorbed in the surface layer. However, as a semi-transparent medium, foams exhibit in-depth absorption of infrared radiation [30]. Because the first cell walls in the top layer only have a fraction of the entire absorption, in-depth absorption can be a main factor for determining $t_{ig}$. [43] To verify this, a simple experiment was performed, as described in [31,32]. The measured intensities, sample thicknesses, and calculated absorption coefficients are displayed in Table 5.

The measurements of the incident radiation on the heat flux meter after passing through the compressed foam sample proved that less than 50% of the radiation was absorbed in the surface layer ranging from 76 µm for PIR-50-P to 104 µm for PUR-50-H.

**Figure 3:**
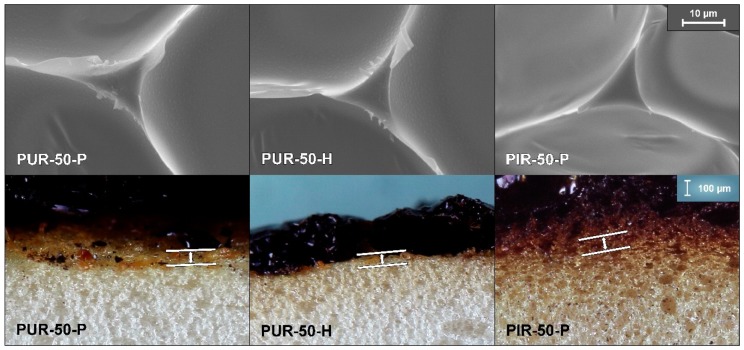
SEM micrograph of a cross section of struts and cell walls for PUR-50-P, PUR-50-H, and PIR-50-P as well as corresponding pyrolysis zones.

Figure 3 shows a SEM micrograph of an average strut and cell walls of PUR-50-P, PUR-50-H and PIR50-P, revealing that the size of a strut is approximately 10 µm and the thickness of a single cell wall less than 1 µm. In-depth absorption is therefore a crucial factor when determining the $t_{ig}$ for all foams tested.

To obtain more reliable results from the estimation of $t_{ig}$, an effective heat flux (${\dot{q}}_{eff}^{\prime \prime }$) for calculating the $t_{ig}$ for RPUFs was defined. Therefore, the absorptance, which describes the fraction of radiation being absorbed within a given distance and which is given by:

(3) $$\alpha =\left(1-e^{-aS}\right),$$

was calculated for each foam for the thickness of the pyrolysis zone contributing to the ignition [30]. A value of 0.10 mm was considered for PUR-50-P, 0.08 mm for PUR-50-H and 0.12 mm for PIR-50-P. The pyrolysis zone contributing to the ignition changes rapidly at the beginning of the cone calorimeter test. While in a first step cell walls and struts absorb the radiation, after short time the absorbing layer consists of liquid pyrolysis products. Since this layer is not homogeneous over the sample surface, thicknesses for calculating the absorptance were roughly estimated by image analysis of images taken of cross sections of quenched foams as shown in Figure 3. The absorptance of the pyrolysis zone ($\alpha_{py}$) for each foam is listed in Table 5. The efficient heat flux was defined as:

(4) $${\dot{q}}_{eff}^{\prime \prime }=\alpha_{py}\cdot {\dot{q}}_{ext}^{\prime \prime }.$$

The $t_{ig}$ values estimated by applying the effective heat flux are listed in Table 4. The respective results still underestimate the actual $t_{ig}$ and have a qualitative character rather than representing a quantitative assessment. It was shown that the investigated foams exhibit a nearly immediate ignition when exposed to a heat flux of 50 kW/m^2^. However, results are of the same order of magnitude and represent a good approximation of the measured $t_{ig}$.

#### 3.3.2. Burning

Results of cone calorimeter measurements for $t_{ig}$, PHRR, $t_{PHRR}$, THR, residue and TML/THR are shown in Table 6. Heat conduction into the bulk of foams was generally low, hence cellular polymers respond very quickly to an imposed heat flux. Therefore, the surface of all tested samples was heated up rapidly after exposure to the external heat flux of the cone calorimeter. This led to a fast supply of pyrolysis products and therefore fast development of sustained burning. All samples underwent distinct charring, and none exhibited structural collapse nor formed a pool fire of liquid pyrolysis products.

**Figure 4:**
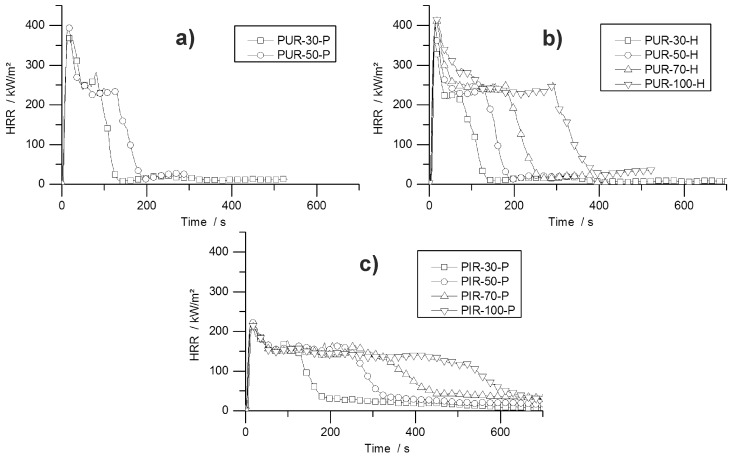
HRR curves for (**a**) pentane-blown PUR, (**b**) water-blown PUR, and (**c**) pentane-blown PIR foams.

HRR curves of all tested materials are shown in Figure 4. The graphs indicate the typical burning behaviour of residue-forming materials [36]. The ignition was followed immediately by the PHRR and the formation of a carbonaceous protective layer. Then, in a second stage, plateau-like behaviour of the HRR occurred, with less intense burning. The length of this plateau depended on the density of the materials and therefore on the amount of combustible material. The burning period was prolonged with increasing sample mass, but had a similar intensity within each group of materials. Minor peaks at the end of the plateau were identifiable for some samples, which were caused by cracking of the residue surface.

The effect of density on burning behaviour has been reported previously for both, flexible and rigid foams [33,44]. The array of curves shown in Figure 4 is also known for char forming samples with different thicknesses. Increasing the sample thickness while keeping the materials’ chemical composition constant leads to a prolonged phase of steady burning. Compared to the burning behaviour of the present foams, the increased sample density causes a similar effect. While keeping the chemical composition of the foam constant, the amount of combustible material is increased [36]. Similar burning behaviour was observed for non-charring polymers like poly(methyl methacrylate) (PMMA). After ignition PMMA exhibits a phase of steady burning with nearly constant HRR the length of which is determined by the sample thickness and therefore by the amount of combustible material [45]. The phenomenon of increased steady burning for foams is based on the same effect, but instead of increasing the sample thickness for a higher amount of combustible material, the density is the parameter varied. While for different sample thicknesses the pyrolysis front consuming the sample is constant, but the burning time increases because the front must travel longer distance, for samples with varying densities the pyrolysis front must travel the same distance but its velocity decreases with increasing density. An experiment on varying properties including the sample thickness revealed the same resulting effect on the mass loss rate of burning polymers. Increased sample thickness did not change the average or peak mass loss rate as long as the sample thickness was large enough for sufficient in-depth absorption [42].

Specific blowing agents act as a gaseous flame retardant in closed cell foams. Trichloro-fluoromethane (CFC-11) acts as a flame inhibitor, has the ability of radical quenching, and has been known to improve the fire performance of foams [41]. Due to its ozone depletion potential, it was banned in the 90 s; consequently, the flammability of RPUF became a major concern. The flammability of blowing agents is still the subject of investigations [46] since blowing agents used nowadays can be non-flammable (e.g., HFC 365/227 93/7 or water) or highly flammable (e.g., mixture of pentane isomers). Nevertheless, PUR-P and PUR-H performed similarly at densities of 30 and 50 kg/m^3^, respectively. The examined cone calorimeter results obtained for both materials exhibited no significant differences. Even though the content of the closed cells is either highly flammable (iso- and cyclo-pentane for pentane-blown foams) or inert (CO_2_ for water-blown foams), the PHRR, average HRR and fire load differs from each other only slightly. Since the pentane adds no more than 3–5 wt % to the overall weight and thus barely any fuel or increased EHC, the fire behaviour of a RPUF is determined mainly by the solid polymer and not by the gas phase [8]. Investigations of pentane- and HFC 365mfc-blown foams have revealed that they do not differ fundamentally from CFC-11- or HCFC 141b-blown foams in their fire behaviour [47,48].

The PHRR of PUR-H increased from 366 kW/m^2^ for PUR-30-H by 17% to 428 kW/m^2^ for PUR-100-H, probably because of the higher amount of combustible material in the surface layer, which led to a higher amount of pyrolysis products and resulted in a higher PHRR for higher-density samples. The higher the amount of pyrolysis product in the top layer, the more heat can be released, leading to an increased PHRR for higher-density materials. Due to the greater fire load of materials with increased mass, the THR also went up with foam density. The char yield changed negligibly with density within the group of all tested PUR-H foams, ranging from 15.7 to 18.5 wt %. This is in accordance with the residual mass obtained from TG, even though residues from TG were slightly higher compared to cone calorimeter results. This is probably due to some oxidative decomposition adding to the pyrolytic decomposition under forced-flaming conditions.

The PHRR of PIR-P samples dropped slightly with increasing density. For these materials the effect of more distinct charring prevailed over the effect of the higher amount of combustible material on the surface for higher-density materials as observed for PUR-H samples. PHRR and average HRR are considerably lower compared to all tested PUR samples, but all exhibited a prolonged time to flameout. The better overall fire performance of PIR foams is caused by the isocyanate ring structure. Positive effects on thermal stability as well as on fire behaviour have been reported previously [41,49,50]. However, it has been found that improved thermal stability does not necessarily lead to better fire performance [51]. This improvement is most probably an effect of better charring, and thus a better protective layer, which is investigated and reported in greater detail later in this paper. With rising density and therefore higher sample weight, THR increased. Compared to PUR-P and PUR-H foams, the fire load was very similar. Even though the HRR was much lower, times to flame out increased. The residue increased with density from 16.7 wt % for PIR-30-P to 24.5 wt % for PIR-100-P, indicating that the superior charring behaviour also results in increasing char yield with increasing density. This is based on the fact that more combustible material in the top layer is able to form a denser char layer protecting underlying material more effectively. In contrast, residue obtained from TG was even higher and similar for all PIR-P foams. In this case, cone calorimeter results differ from TG results, since macroscopic effects like the formation of protective layer are not recognized well by TG. EHC did not change significantly for any of the foams.

**Figure 5:**
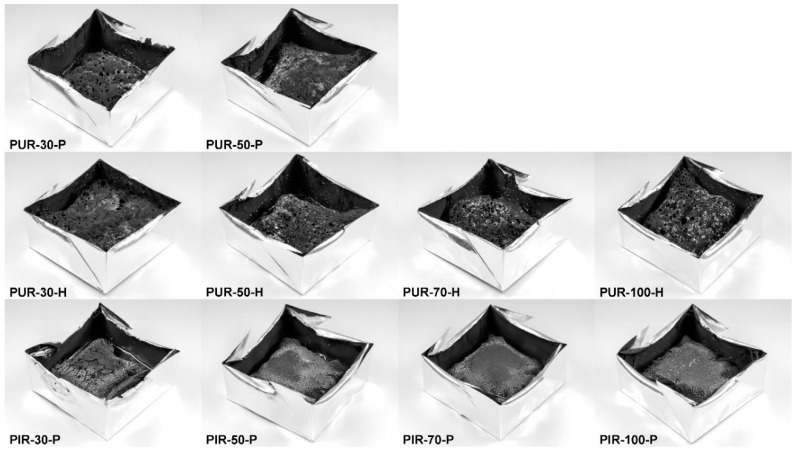
Cone calorimeter residues of PUR-P, PUR-H, and PIR-P foams.

**Figure 6:**
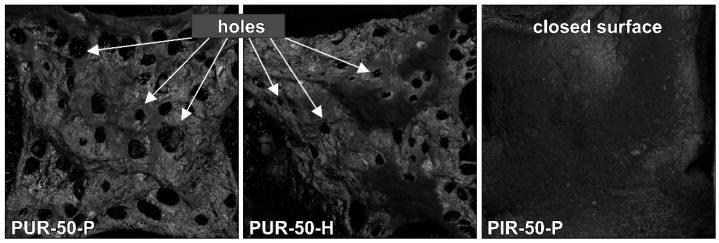
Macro-photograph of residue surfaces of PUR-50-P, PUR-50-H, and PIR-50-P.

Residues of the cone calorimeter experiments are displayed in Figure 5. There are fundamental differences in the residues’ quality on a macroscopic level, even though SEM investigation of the char showed that the surface of every residue was closed and compact. The protective layers formed during the combustion of the specimen were of similar quality within every group of materials, independent of their density. While PUR-P and PUR-H developed a residue in which holes were visible, the residues of PIR-P had a closed and compact surface. Figure 6 shows the surface of the samples with a density of 50 kg/m^3^, which were quenched at 50 wt % of TML. Since PUR-P and PUR-H foams exhibited a minor char yield, and the char formed during combustion seemed to have a low viscosity as it was bubbling during combustion, their residues had an open surface and therefore did not cover the whole sample. The char of PUR-P and PUR-H foams was fragile and brittle affording only minor protection since their surface was not closed. PIR-P samples showed a closed surface residue covering the whole sample, due to their increased char yield. Directly after ignition, a closed and compact protective layer was formed. After flame out the residue was stable and dense. This correlates well with the observations of PHRR and HRR during the phase of steady burning, since PHRR and average HRR were significantly lower for PIR-P foams.

### 3.4. Temperature Development Inside Burning Specimens

**Figure 7:**
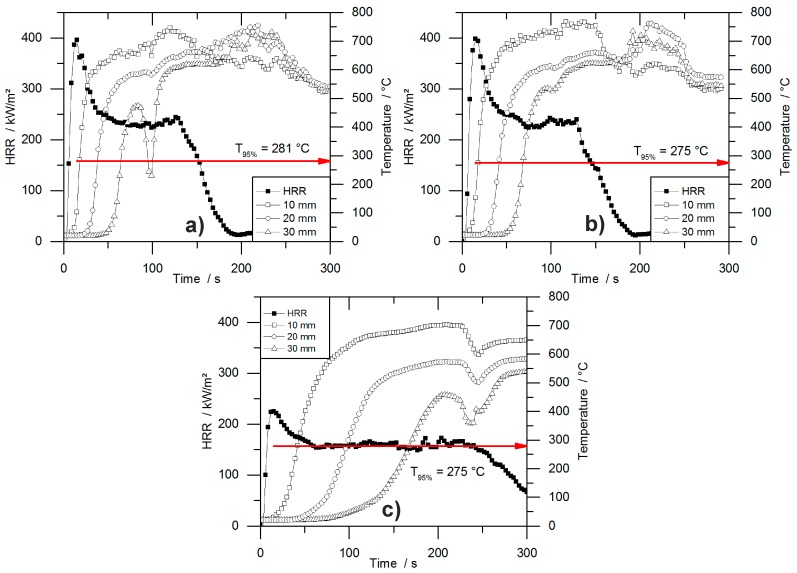
Heat release rate curve and temperature development inside a burning specimen of (**a**) PUR-50-P (**b**) PUR-50-H, and (**c**) PIR-50-P.

During the cone calorimeter test, the temperature development inside burning specimens was monitored at depths of 10, 20, and 30 mm. The HRR curve and the temperature development inside the specimens were plotted over time and are displayed in Figure 7 for PUR-50-P, PUR-50-H, and PIR-50-P. The graphs show the excellent thermal insulating properties of the foams. Even though the PHRR of the burning top layer was already reached after 16.5 s for both materials, the temperature measured at a depth of 10 mm remained low. As soon as the pyrolysis front approached the thermocouple there was a steep rise in temperature. By the time the T_95%_ was reached at a depth of 10 mm, the protective layer on the surface had already formed and HRR began to drop to the steady burning HRR. After passing the thermocouple, the foam was consumed by the fire such that the thermocouple was partially covered by residue and partially exposed. Thus, it was measuring the temperature of the flame, which is the reason for the erratic temperature signal above 500 °C. The drop in temperature displayed in Figure 7b for the 30 mm signal was caused by cracking of the residue. Something similar happened in Figure 7c.

Comparing the temperature signals to those of PIR-50-P, the times to reach T_95%_ at each depth of measurement increased significantly for PIR-50-P. This is caused by the lower MLR under cone calorimeter test conditions and by the better fire performance of this material. For PIR foams those observations correlate well with the reduced PHRR, reduced average HRR, and their increased char yield, and therefore improved protective layer compared to PUR-P and PUR-H foams. Additionally, approaching the first thermocouple at a depth of 10 mm for the pyrolysis front still takes longer than for PUR foams. This also correlates well with the results from the cone calorimeter like PHRR, average HRR and residue. The graphs for temperature signal in Figure 7c are smoother, since the thermocouple was fully covered with char.

**Figure 8:**
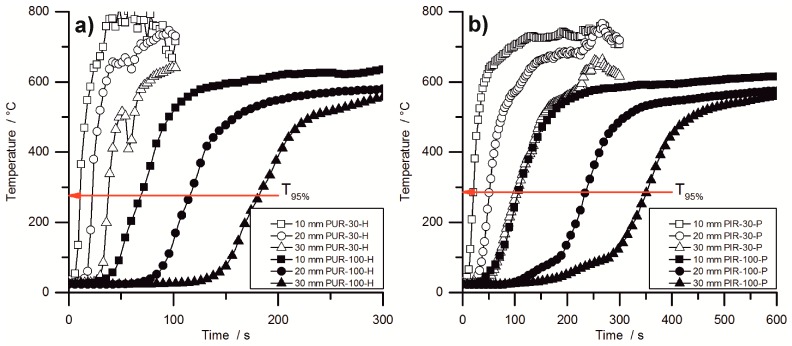
Temperature development inside (**a**) PUR-H and (**b**) PIR-P foams at densities of 30 and 100 kg/m^3^.

Figure 8 shows the temperature development inside burning specimens for PUR-H and PIR-P foams, for the lowest and highest densities, respectively. The slopes of the graphs are investigated by determining the maximum of the first derivation, which is defined here as the maximum heating rate (MHR). In the graphs, it is clear that the slope of the temperature curves is steeper with lower density. Figure 8 makes clear that the onset of temperature rise at a depth of 10 mm is delayed crucially by increasing the density. Although $t_{PHRR}$ obtained from cone calorimeter test is hardly affected by the density, the velocity of the pyrolysis front is. This proves that the formation of the protective carbonaceous layer depends only on the amount of combustible material that is consumed by the fire. The increasing density of the foam leads to an earlier formation of char. The MHRs shown in Table 7 were recorded within the first 100 s for PIR-30-P, while the pyrolysis front passed the last thermocouple only after more than 300 s for PIR-100-P. Thermocouple signals for PIR-100-P at depths of 20 mm and 30 mm showed an obvious rise in temperature from ambient temperature to approximately 100 °C, which was caused by conduction of heat during combustion through the foam. Following this, a slight kink in the signal occurred and subsequently a steeper increase of temperature took place, which can be attributed to the approach of the pyrolysis front. The drop of temperature as displayed for the 30 mm signal of PUR-30-H was caused by cracking of the residue.

Table 7 contains the maxima of heating rates (MHR) deduced from the temperature curves as well as the time at which the MHR occurred ($t_{MHR}$) for every depth of measurement (10, 20 and 30 mm). In general, within each group of materials the MHR exhibited a decrease with increasing density and with increasing depth of measurement. Most probably, this is an effect of the cone calorimeters’ decreasing heat flux at increasing distance from the thermocouple, but also a result of the chars’ growing thickness and therefore better protective layer. Lower MHR and longer $t_{MHR}$ indicate a better protective layer with increasing depth of measurement, but not necessarily a lower velocity of the pyrolysis front since there is an overlapping effect of heat conduction through the sample. Therefore the MHR decreased at depths of 20 mm and 30 mm compared to 10 mm.

PUR-P and PUR-H foams exhibited the highest heating rates with respect to density. MHR for pentane-blown foams were slightly higher at 10 mm, which is probably an effect of the highly combustible blowing agent, although the overall fire performance determined with the cone calorimeter was not affected compared to water-blown RPUF. However, compared to PUR foams, a significant difference was observed for PIR-P. The MHR that was measured at a depth of 10 mm decreased from 89 °C/s for PUR-30-H by 52% to 43 °C/s for PIR-30. This phenomenon is a consequence of the better fire performance of PIR-P foams under forced flaming conditions, which has been discussed previously. PIR foams generally exhibited the lowest MHR and longest $t_{MHR}$.

In general, the measured increase in temperature slowed down with rising density and increasing depth of measurement. Furthermore, the maximum heating rates decreased and the times to maximum heating rates increased. While this is probably an effect of heat conduction through the sample, and is not significant for determining the actual velocity of the pyrolysis front, it indicates crucial variation in the burning behaviour of the foam with increasing density. For low-density foams the temperature signal exhibited a steep increase as soon as the pyrolysis front approached the thermocouple. In contrast, for high-density foams a more moderate rise was observed, including a minor rise in the temperature inside the specimen prior to pyrolysis, caused by thermal conduction through the bulk of the foam. This implies a shift of the burning behaviour toward the behaviour of a solid, non-cellular material with increasing density.

**Figure 9:**
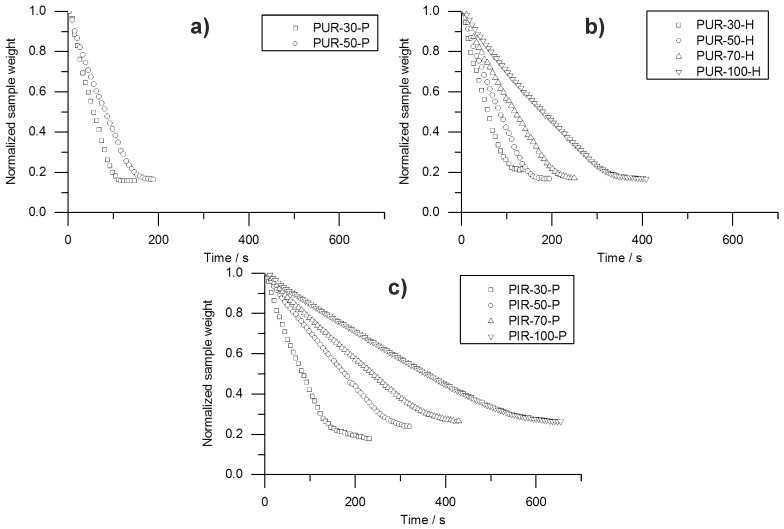
History of normalized sample weight for (**a**) PUR-P, (**b**) PUR-H and (**c**) PIR-P foams.

**Figure 10:**
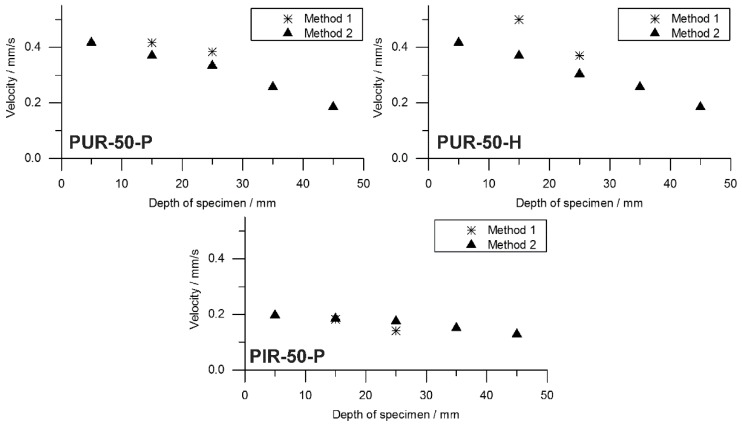
Comparison of pyrolysis front velocity deduced from thermocouple and mass loss data for PUR-50-P, PUR-50-H, and PIR-50-P.

Information on the velocity of the pyrolysis front can be taken from the temperature development measured inside the burning specimens. The T_95%_ obtained from TG was consulted to define the time at which the pyrolysis front reached the depth of temperature measurement. A prolonged phase of steady burning in the cone calorimeter test while the specimens’ size is held constant is associated with a decreased velocity of the pyrolysis front. To investigate this phenomenon, the velocity was calculated by considering the times to T_95%_. Hence, velocity was determined from sample depths of 10 to 20 mm and from 20 to 30 mm. This was defined as Method 1. Interestingly, the mass loss obtained from the cone calorimeter test indicates linear behaviour throughout the test until flame out. The normalized sample weight plotted over time is shown for each tested material in Figure 9. Measurement data start at $t_{ig}$ and were recorded until briefly after flame out. For each material, a kink in the mass loss curve was observed. This marks the transition from flaming combustion and pyrolytic decomposition to afterglow of the carbonaceous residue. The normalized sample weights were fitted starting from $t_{ig}$ to flame out and the resulting pyrolysis front velocity was calculated. Therefore, the assumption was made that the decrease of mass is proportional to the decrease of volume and that the amount of residue formed during combustion was consistent over time. This was defined as Method 2. Results from Method 1 and Method 2 were compared and plotted over depth of specimen. This comparison was done for each foam at a density of 50 kg/m^3^ and is shown in Figure 10.

Both Method 1 and Method 2 revealed decreasing pyrolysis front velocities for all foams. As already discussed above, this is probably an effect of the increasing thickness of the char layers and the decreasing intensity of radiation from the conical heater with greater distance [34]. Additionally, the appearance of the PHRR directly following ignition at the very beginning of the cone calorimeter measurement gives evidence of the thickest pyrolysis zone or the fastest velocity of the front, respectively [35]. While the cone calorimeter mass loss data indicates a constant velocity of the pyrolysis front (Figure 9), thermocouple measurements actually prove that the velocity decreases slightly with increasing depth of measurement. The reason for the mass loss data remaining constant may be additional smouldering of the residue already during flaming combustion as experienced in a previous study of PIR and phenolic foams [52]. Nevertheless, the two signals show good correlation of Method 1 and Method 2 for PUR-50-P and PUR-50-H, both of which burned down more erratically than PIR foams. In fact, there was an even stronger correlation between the results of PIR-50-P since the burning behaviour was smoother.

The average velocities of the pyrolysis fronts of foams with a density of 50 kg/m^3^ are listed in Table 8. The results were averaged from depths of 10 to 30 mm for both methods in order to cover the same area of measurement. Method 2 gives a decrease of velocity of 13% for PUR-50-P and 23% for PUR-50-H, respectively. In contrast, the velocity of PIR-50-P increased by 13% with respect to Method 1. The reasons for this are probably the limitations of both methods. Results of Method 1 suffered from an unevenly burning sample whose decomposing surface layer is not a perfect plane. This causes the pyrolysis front to reach the thermocouples at various times. Pyrolysis front velocity as derived from mass loss in the cone calorimeter test is subject to system-related scatter effects since the samples weight is very low. Comparing the velocity of the investigated foams with the one of a bulk polymer elucidates how quick the cellular materials are consumed by the fire due to their low density. For PMMA, as a non-charring polymer exhibiting a thermally thick burning behaviour, an average velocity of the pyrolysis front of 0.025 mm/s (1.51 mm/min) was measured [53]. For an epoxy resin (hardly charring) and its layered silicate nanocomposite, which formed a protection layer, values of 0.012–0.023 mm/s (0.7–1.4 mm/min) and 0.008–0.012 (0.5–0.7 mm/min) were measured, respectively [54]. Pyrolysis front velocities of the investigated foams exceeded those of bulk polymers by more than 10-fold.

### 3.5. Pyrolysis Zone

**Figure 11:**
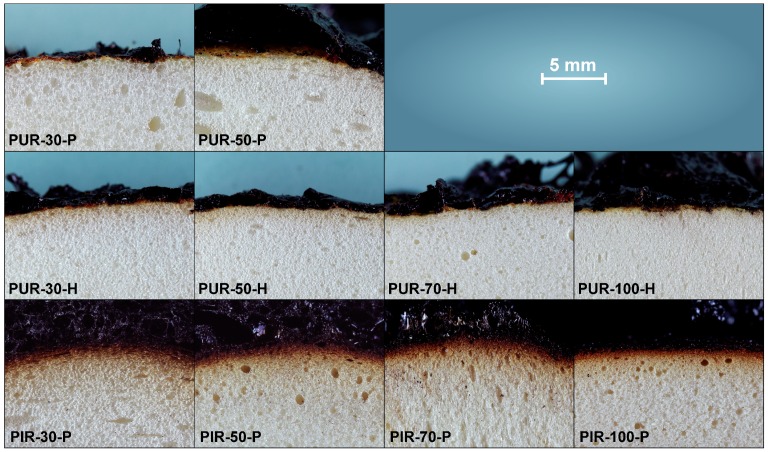
Macro photographs of cross sections of quenched samples.

Macro-photographs of the quenched samples are shown in Figure 11. Pyrolysis was interrupted by quenching in liquid nitrogen to preserve the samples’ structure at the point of 50 wt % mass loss. PUR-P and PUR-H foams exhibit a completely undamaged foam structure underneath the pyrolysis front and do not show any discoloration. In contrast, PIR-P foams show a small zone of discoloration indicating thermal decomposition underneath the char. However, the thickness of the discoloured zone does not change significantly with density.

**Figure 12:**
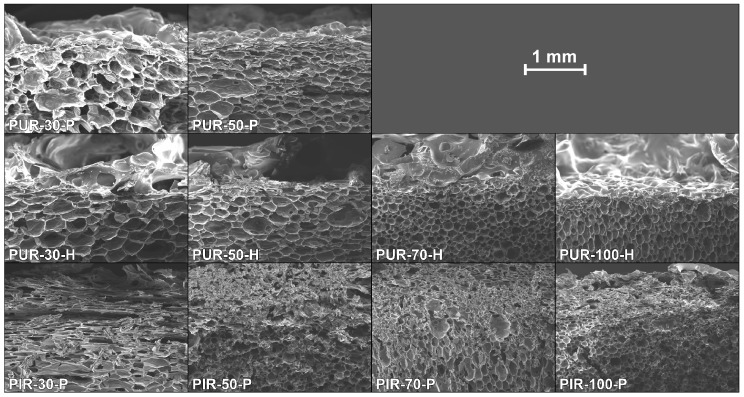
SEM micrographs of cross sections of quenched samples.

Figure 12 displays SEM images of cross sections of cone calorimeter specimens of all of the samples tested. Despite the difference in densities, a similar behaviour was revealed within every group of materials. Here, a distinction must be made between the PUR and the PIR foams. PUR-P and PUR-H exhibited a definite pyrolysis front with a clear boundary between the unchanged foam structure and the charred residue. This can be interpreted as a result of the very good thermal insulating properties of the foam and the velocity of the pyrolysis front. Since the heat conduction through the sample is very low and the rate of mass loss is high, the structure of the foam did not undergo any kind of morphological change until the material was pyrolyzed and consumed by the fire. The foams’ morphology was then lost once the pyrolysis front passed the sample. This is coherent with an earlier study [55].

**Figure 13:**
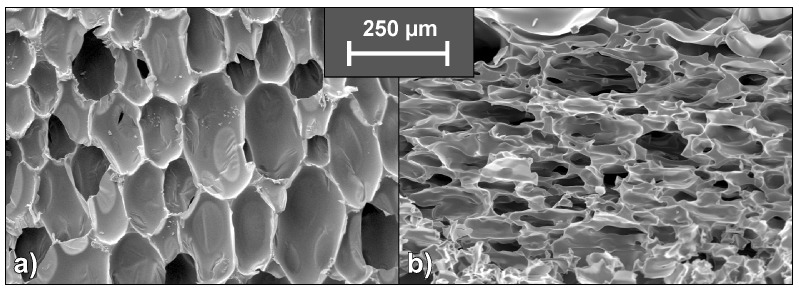
SEM micrographs of PIR-70-P, structure of the (**a**) foam and (**b**) residues.

Examination of the cross sections of PIR-P foams showed different behaviour. Instead of a clear pyrolysis front, a pyrolysis zone was observed. It is assumed that the inherent char yield of PIR foams, which is different from PUR, is the reason for this phenomenon. The SEM micrographs in Figure 13 indicate that the morphology of the foam is retained in the residue to some degree or that at least the char is built up in a foam-like structure. Thus, under forced-flaming conditions PIR-P foams yield a more effective protective layer than do PUR foams with respect to their thermal insulating properties.

**Figure 14:**
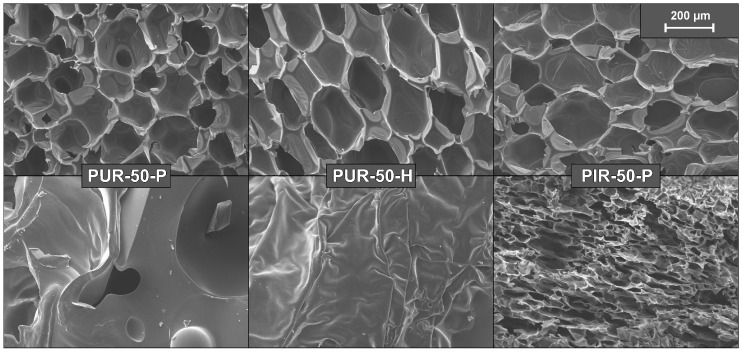
SEM micrographs of the structure of PUR-50-P, PUR-50-H, and PIR-50-P foam and residue.

Figure 14 displays the undamaged foam structure of PUR-50-P, PUR-50-H, and PIR-50-P as well as the structure of their residues after quenching. The residue was thin and brittle with a closed surface, offering meagre protection to the underlying material. It can be seen clearly that part of the residue of PIR-P foam is hollow, with a kind of a cellular structure. In general, the residue formed by the PIR foam was more stable, denser and thicker compared to PUR foams. This phenomenon is proposed to be the main reason for the superior fire performance and is worth examining in greater detail and on a larger scale.

## 4. Conclusions

Rigid pentane- and water-blown polyurethane foams as well as pentane-blown polyiso-cyanurate foams were investigated and their fire phenomena were determined in detail. While TG measurements exhibited similar pyrolysis temperatures for PUR-P, PUR-H, and PIR-P foams, overall thermal stability turned out to be best for PIR-P, which also yielded the highest amount of char, contributing to their improved fire performance. For each group of materials, LOI increased with density and was highest for PIR-P foams.

Under forced-flaming conditions, all materials tested ignited immediately after exposure to the external heat flux. PHRR occurred after ignition, followed by the formation of a carbonaceous protective layer and a significant decrease in HRR, as known for char-forming materials. A phase of steady burning followed, the length of which was determined by the density and therefore by the amount of combustible material for each foam. Cone calorimeter measurements underlined the superior overall fire performance of PIR foams compared to PUR foams. PHRR as well as steady burning HRR decreased significantly for PIR-P. The THR increased with density for each group of materials caused by the rising amount of combustible material. The fire load of all materials of same density did not differ significantly, since the lower HRR of PIR foams was compensated by longer burning times. EHC was similar for all materials tested. The calculation of $t_{ig}$ in the cone calorimeter with the established formula for thermally thick materials correlated poorly with the measurements. For more reliable results, a correction factor was defined, yielding an ${\dot{q}}_{eff}^{\prime \prime }$ that improved the estimation of $t_{ig}$ for RPUFs under cone calorimeter test conditions.

Thermocouples inserted into the burning specimens revealed the temperature development inside the materials under forced flaming conditions. It was shown that for low-density foams the temperature increased suddenly when the pyrolysis front passed. The temperature of the underlying material remained low. With increasing density, the rise of temperature was moderated and heating rates decreased. Additionally, the effect of thermal conduction through the sample became visible in the thermocouple signal, indicating a shift in burning behaviour towards solid materials.

Quenching of the burning cone calorimeter specimens and investigation of cross sections revealed a clear pyrolysis front for all investigated PUR foams. None of the foams collapsed or exhibited a tendency to dripping. The structure of the rigid foams was retained until the pyrolysis front passed and the foam was consumed by the fire. This structural integrity is a result not only of the cross-linked chemical structure, but also the excellent insulating properties. The morphology of the foams underneath the pyrolysis front was unchanged. Despite differences in densities, every material had similar behaviour except PIR foams. Those materials had a superior char yield and their cellular structure was retained in the charred residue to some extent, contributing to improved protective properties of the char layer.

This paper gives detailed insight into the fire phenomena of pentane- and water-blown polyurethane as well as pentane-blown polyisocyanurate polyurethane foams.

## Figures and Tables

**Table 1:** Foams investigated.

Material	Chem. Structure	Blowing Agent	Density (kg/m^3^)
PUR-30-P	PUR	pentane + water	34.9 ± 0.3
PUR-50-P	PUR	pentane + water	52.2 ± 0.7
PUR-30-H	PUR	water	30.7 ± 0.5
PUR-50-H	PUR	water	52.0 ± 0.1
PUR-70-H	PUR	water	73.9 ± 0.9
PUR-100-H	PUR	water	107.8 ± 1.7
PIR-30-P	PIR	pentane + water	33.7 ± 2.5
PIR-50-P	PIR	pentane + water	55.9 ± 0.3
PIR-70-P	PIR	pentane + water	68.6 ± 4.0
PIR-100-P	PIR	pentane + water	110.7 ± 5.3

**Table 2:** Results of foam characterization.

Material	λ @10 °C (mW/m·K)	Compressive Strength (kPa)
PUR-30-P	29.6 ± 0.2	195 ± 9
PUR-50-P	25.7 ± 0.2	338 ± 4
PUR-30-H	29.8 ± 0.2	170 ± 8
PUR-50-H	27.7 ± 0.2	343 ± 14
PUR-70-H	28.5 ± 0.2	424 ± 10
PUR-100-H	26.6 ± 0.2	1025 ± 23
PIR-30-P	28.6 ± 0.2	348 ± 2
PIR-50-P	28.2 ± 0.2	355 ± 4
PIR-70-P	25.1 ± 0.2	369 ± 11
PIR-100-P	29.8 ± 0.2	1091 ± 28

**Table 3:** Results of TG and LOI measurements.

Material	T_95%_ (°C)	T_max_ (°C)	Residue (wt %)	Avg. Residue (wt %)	LOI (vol %)
PUR-30-P	274	290	19.9	19.8 ± 0.1	19.5 ± 0.3
PUR-50-P	275	291	19.7		20.4 ± 0.2
PUR-30-H	277	296	21.5	20.4 ± 1.1	20.1 ± 0.2
PUR-50-H	281	292	21.5		20.5 ± 0.2
PUR-70-H	276	291	19.3		21.0 ± 0.2
PUR-100-H	275	290	19.3		21.3 ± 0.1
PIR-30-P	279	338	26.7	27.4 ± 0.7	21.5 ± 0.2
PIR-50-P	279	339	27.8		22.4 ± 0.2
PIR-70-P	289	340	28.0		22.9 ± 0.2
PIR-100-P	293	339	27.1		23.1 ± 0.1

**Table 4:** Specific heat capacity as well as calculated, measured, and corrected $t_{ig}$ for foams with a density of 50 kg/m^3^.

Material	$t_{ig}$ (s) Measured	c (J/kg K)	$t_{ig}$ (s) Calculated	$t_{ig}$ (s) Calculated
PUR-50-P	3 ± 1	1304 ± 52	0.14	0.90
PUR-50-H	2 ± 1	1315 ± 47	0.15	1.30
PIR-50-P	3 ± 1	1313 ± 40	0.16	0.79

**Table 5:** Measured intensities (*I*/*I*_0_), sample thicknesses (*S*), absorption coefficients (*a*), and absorptance of pyrolysis zone ($\alpha_{py}$) for every foam with a density of 50 kg/m^3^.

Material	*I*/*I*_0_ (%)	*S* (µm)	*a* (mm^−1^)	$\alpha_{py}$
PUR-50-P	63 ± 4	91 ± 13	5067	0.40
PUR-50-H	58 ± 1	104 ± 9	5272	0.34
PIR-50-P	69 ± 2	76 ± 11	4900	0.44

**Table 6:** Results of cone calorimeter tests.

Sample	$t_{ig}$ (s)	PHRR (kW/m^2^)	$t_{PHRR}$ (s)	THR (MJ/m^2^)	Residue (wt %)	TML/THR (MJ/m^2^g)
PUR-30-P	2 ± 1	368 ± 3	15.0 ± 1.0	27 ± 2	15.5 ± 0.4	2.0 ± 0.1
PUR-50-P	3 ± 1	405 ± 7	16.5 ± 1.5	40 ± 2	16.4 ± 0.0	1.9 ± 0.1
PUR-30-H	2 ± 1	366 ± 14	16.5 ± 4.5	27 ± 2	18.5 ± 3.7	2.0 ± 0.1
PUR-50-H	2 ± 1	395 ± 1	16.5 ± 1.5	41 ± 2	15.7 ± 1.0	1.9 ± 0.1
PUR-70-H	3 ± 1	417 ± 1	16.5 ± 1.5	57 ± 1	16.9 ± 0.1	1.9 ± 0.1
PUR-100-H	4 ± 1	428 ± 13	19.5 ± 1.5	87 ± 1	16.5 ± 0.0	1.9 ± 0.1
PIR-30-P	2 ± 1	234 ± 6	13.5 ± 1.5	28 ± 1	16.7 ± 0.9	2.1 ± 0.1
PIR-50-P	3 ± 1	226 ± 1	16.5 ± 1.5	47 ± 1	22.8 ± 1.1	2.1 ± 0.1
PIR-70-P	3 ± 1	226 ± 9	15.0 ± 1.0	55 ± 4	23.5 ± 2.2	2.1 ± 0.1
PIR-100-P	4 ± 1	219 ± 5	18.0 ± 1.0	81 ± 3	24.5 ± 1.8	2.0 ± 0.1

**Table 7:** Maximum heating rates at depths of 10, 20, and 30 mm for all tested materials.

Sample	10 mm in Depth		20 mm in Depth		30 mm in Depth	
	MHR (°C/s)	$t_{MHR}$ (s)	MHR (°C/s)	$t_{MHR}$ (s)	MHR (°C/s)	$t_{MHR}$ (s)
PUR-30-P	97	14	62	24	52	44
PUR-50-P	84	21	45	45	40	73
PUR-30-H	89	15	76	26	55	42
PUR-50-H	75	21	50	41	39	67
PUR-70-H	38	44	31	75	25	112
PUR-100-H	24	71	22	127	16	195
PIR-30-P	43	20	11	50	10	90
PIR-50-P	22	44	11	98	10	172
PIR-70-P	14	63	8	141	9	239
PIR-100-P	8	107	7	231	7	367

**Table 8:** Average pyrolysis front velocities of PUR-50-P, PUR-50-H, and PIR-50-P.

	PUR-50-P	PUR-50-H	PIR-50-P
	Avg. Velocity (mm/s)	Avg. Velocity (mm/s)	Avg. Velocity (mm/s)
Method 1	0.40	0.44	0.16
Method 2	0.35	0.34	0.18
